# Blood‐based detection of lung cancer using cysteine‐rich angiogenic inducer 61 (CYR61) as a circulating protein biomarker: a pilot study

**DOI:** 10.1002/1878-0261.13099

**Published:** 2021-10-03

**Authors:** Lucija Ac\ˇkar, Swaantje Casjens, Antje Andreas, Irina Raiko, Thomas Brüning, Maria Geffken, Sven Peine, Jens Kollmeier, Georg Johnen, Kai Bartkowiak, Daniel Gilbert Weber, Klaus Pantel

**Affiliations:** ^1^ Department of Tumor Biology University Medical Center Hamburg‐Eppendorf Germany; ^2^ Institute for Prevention and Occupational Medicine of the German Social Accident Insurance Institute of the Ruhr University Bochum (IPA) Germany; ^3^ Department of Transfusion Medicine University Medical Center Hamburg‐Eppendorf Germany; ^4^ Lungenklinik Heckeshorn Helios Klinikum Emil von Behring Berlin Germany

**Keywords:** biomarker, cancer, CYR61, liquid biopsy, lung cancer, plasma

## Abstract

Lung cancer is the most often diagnosed cancer and the main cause of cancer deaths in the world compared with other tumor entities. To date, the only screening method for high‐risk lung cancer patients is low‐dosed computed tomography which still suffers from high false‐positive rates and overdiagnosis. Therefore, there is an obvious need to identify biomarkers for the detection of lung cancer that could be used to guide the use of low‐dosed computed tomography or other imaging procedures. We aimed to assess the performance of the protein cysteine‐rich angiogenic inducer 61 (CYR61) as a circulating biomarker for the detection of lung cancer. CYR61 concentrations in plasma were significantly elevated in 87 lung cancer patients (13.7 ± 18.6 ng·mL^−1^) compared with 150 healthy controls (0.29 ± 0.22 ng·mL^−1^). Subset analysis stratified by sex revealed increased CYR61 concentrations for adenocarcinoma and squamous cell carcinoma in men compared with women. For male lung cancer patients versus male healthy controls, the sensitivity was 84% at a specificity of 100%, whereas for females, the sensitivity was 27% at a specificity of 99%. The determination of circulating CYR61 protein in plasma might improve the detection of lung cancer in men. The findings of this pilot study support further verification of CYR61 as a biomarker for lung cancer detection in men. Additionally, CYR61 is significantly elevated in women but sensitivity and specificity for CYR61 are too low for the improvement of the detection of lung cancer in women.

## Graphical abstract

In this study, we assessed the performance of circulating CYR61 concentration as a biomarker in plasma of patients with lung cancer (LC) by ELISA. Subset analysis stratified by sex revealed increased CYR61 concentrations for adenocarcinoma and squamous cell carcinoma in men compared with women. Our data support the further verification of CYR61 as a biomarker for lung cancer detection in men. Additionally, CYR61 was significantly elevated in women, but sensitivity and specificity for CYR61 were too low for improvement of LC detection.

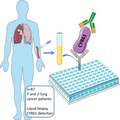

**Abbreviations**
AUC: area under the curve
CI: confidence interval
CYR61: cysteine‐rich angiogenic inducer 61
ELISA: enzyme‐linked immunosorbent assay
IARC: International Agency for Research on Cancer
IQR: interquartile range
PBMC: peripheral blood mononuclear cells
YI: Youden's index

## 1 Introduction

Lung cancer (LC) is the most often diagnosed cancer and the main cause of cancer deaths in the world compared with other tumor entities (1.8 million deaths in 2020, WHO World Cancer Report) [1]. Non‐small‐cell lung cancer (NSCLC) and small cell lung cancer (SCLC) account for approximately 85% and 15% of all diagnosed LC, respectively [2]. In NSCLC, the most frequently diagnosed histological subtypes are adenocarcinoma (ADC, 40–50% of diagnoses) and squamous cell carcinoma (SCC, 20–30% of diagnoses) [3].

The only screening method seeming suitable for high‐risk LC patients is low‐dosed computed tomography (LDCT) [4]. The use of appropriate multivariable risk prediction models significantly affects the sensitivity and specificity of LDCT. For example, the US National Lung Screening Trial (NLST) involved current and former heavy smokers (≥ 30 packs per year of cigarette smoking history; former smokers were included if they quit smoking ˂ 15 years before) aged 55–74 years. The participants underwent three annual rounds of LDCT or single‐view chest radiography, and LC was identified at baseline in 1.03% of participants [5]. The UK Lung Cancer Screening (UKLS) trial involved participants with a 5‐year LC risk of ≥ 5% according to the Liverpool Lung Project (LLP) multivariable risk prediction model, together with an age of 50–75 years. Participants underwent LDCT‐based screening or no screening, and 1.7% of participants were identified with LC [5]. In the NELSON trial, participants were current and former heavy smokers (≥ 30 packs per year) aged 55–75 who underwent several rounds of LDCT‐based screening or no screening and 0.9% of participants were identified with LC [5]. Despite encouraging recent results, LDCT suffers still from high false‐positive rates and overdiagnosis [6]. Moreover, intensive evaluations following a positive LDCT are associated with more procedure‐related adverse events, higher mean radiation exposure and higher mean health expenditure [7].

Thus, there is an obvious need to identify biomarkers for the detection of LC that could be used to guide the use of LDCT or other imaging procedures. Incorporation of blood biomarkers (‘liquid biopsy’) into the prediction models might have the potential to increase precision of LDCT‐based LC screening.

Mutations and gene methylation related to LC, such as mutations in *TP53, EGFR, BRAF, ERBB2, PDGFRA* and *KRAS* or methylation of *PTGER4/SHOX2*, were previously detected in plasma or cerebrospinal fluid of NSCLC and SCLC patients, both in early and late stages [4, 8, 9, 10]. Moreover, circulating tumor cells (CTCs) have been investigated for early LC detection [11] but a recent validation trial showed that despite initially encouraging results [12], CTCs captured by the ISET method failed to provide sufficient accuracy [13]. New members of the liquid biopsy family applied for LC detection include tumor‐macrophage fusion cells (TMF) [14], circulating microRNAs (miRNAs) [15], tumor‐educated platelets [16], and extracellular vesicles (EVs) [17]. Proteomic profiling of EVs showed that EVs are packed in a tumor‐specific manner [18, 19]. Exosome number or presence of certain miRNAs can also indicate the presence of NSCLC [20].

In the present study, we assessed the potential value of CYR61 in plasma as a biomarker for the detection of LC. CYR61 belongs to the CCN family which has six members: CYR61 (CCN1), CTGF (CCN2), NOV (CCN3), WISP‐1 (CCN4), WISP‐2 (CCN5), and WISP‐3 (CCN6). These proteins have modular architecture which consists of an N‐terminal signal sequence followed by domains with sequence similarity to insulin‐like growth factor‐binding protein, von Willebrand factor C, thrombospondin type 1, and a cysteine knot at the C terminus. The exception is WISP‐2, which lacks the C‐terminal region [21, 22]. CCN proteins are secreted extracellular proteins associated with matrix, and they are involved in both, internal and external cellular signaling [21]. CYR61 is involved in cell adhesion, cell migration, stimulation of chemotaxis, enhancement of growth factor‐induced DNA synthesis, cell survival, and angiogenesis [23].

The role of CYR61 in LC is still under investigation with controversial results. Several authors reported that mRNA levels of *CYR61* were decreased in lung tumors compared with normal matched lung tissue [21, 24, 25] and its overexpression decreases colony formation, reduces proliferation, and leads to growth arrest through the p53‐c‐myc‐beta catenin pathway in LC cells [25, 26, 27]. In contrast, CYR61 has been shown to promote epithelial‐to‐mesenchymal transition, cell viability, tumor growth, and metastasis [27, 28, 29, 30].

Elevated CYR61 concentrations have been found in patients with asbestos‐related diseases [31], and CYR61 is also a tumor‐promoting factor in breast, ovarian, and gastric cancer as well as in gliomas and pancreatic neuroendocrine tumors [22, 32, 33, 34, 35, 36, 37, 38, 39].

To the best of our knowledge, this is the first report on circulating CYR61 protein in LC. We compared circulating CYR61 concentrations in plasma of patients with LC and healthy controls, and our pilot study provided the first evidence for a sex‐dependent effect of CYR61 on the detection of LC.

## 2 Materials and methods

A detailed description of the methods and experiments is provided in the online Data Supplement.

### 2.1 Cell lines and culture conditions

The disseminated tumor cell (DTC) cell line from the bone marrow of a LC patient LC‐M1 was generated 1994 and authenticated by Klaus Pantel [40, 41]. LC‐M1 was authenticated using a keratin/vimentin double staining in May 2015 and was essentially cultivated as described before [42]. Previous reports describe the authentication, generation and the attributes of the generated DTC cell lines [40, 41, 43, 44, 45]. LC‐M1 was cultured at 37 °C in a humidified environment with 5% of carbon dioxide and 10% of oxygen. Nitrogen served for the adjustment of the oxygen concentration. LC‐M1 was cultured in Roswell Park Memorial Institute (RPMI) 1640 medium with added 2 mm L‐glutamine, 10 mg·L^−1^ insulin, 5.5 mg·L^−1^ transferrin (all from Life Technologies, Darmstadt, Germany), 10% fetal bovine serum, 10 µg·L^−1^ human basic fibroblast growth factor (b‐FGF, Miltenyi Biotec, Bergisch Gladbach, Germany), and 50 µg·L^−1^ epidermal growth factor (EGF; Miltenyi Biotec).

For the authentication of the cell lines, multiplex cell authentication (SNP‐Profiling) by Multiplexion, Heidelberg, Germany, was performed. The last authentication for H1395 was done in May 2015 and for H1299 in June 2018. HCC‐366 was obtained from the German Collection of Microorganisms and Cell Cultures GmbH (DSMZ, Braunschweig, Germany) in September 2018. H1993 and H1395 were kindly provided by Carsten Müller‐Tidow and H1299 by Harriet Wikman.

The cultivation of H1395, H1299, HCC‐366, and H1993 was performed in RPMI 1640 with 10% fetal bovine serum and 2 mm L‐glutamine (all from Life Technologies), and all cell lines were kept at 37 °C in a humidified environment in the presence of 5% CO_2_. The analyzed samples here were generated within 6 months after revitalization of the cell lines.

### 2.2 Cell harvest and sample procurement for western blot

Prior to cell harvest, the cells were washed with 37 °C prewarmed phosphate‐buffered saline three times. Subsequently, the cells were collected in 300 µL of lysis mix (9.8 m urea, 15 mm EDTA, 30 mm Tris) per 75‐cm^2^ cell culture flask. The cells were disrupted on ice using the using the ultrasonic device UP50H (Hielscher, Teltow, Germany) by 3 identical steps (amplitude 100%; 10 s). After incubation at room temperature for 1 h, the cell extract was clarified from insoluble debris by centrifugation (15 000 ***g*** at room temperature for 5 min) and collection of the supernatant.

The protein concentration was determined using the Pierce BCA Protein Assay Kit (Pierce, Rockford, TN, USA) according to the manufacturer’s instructions and using BSA as the standard. The samples were stored at −80 °C.

### 2.3 SDS/PAGE and western blot

For the separation of the proteins, a Laemmli buffer system and 10% polyacrylamide gels were used. For the gel runs, either the Protean II xi cell (Bio‐Rad, Hercules, CA, USA) or the Novex XCell Sure‐Lock mini system (Invitrogen, Groningen, the Netherlands) was used. From each cell extract, 20 µg of samples was diluted in SDS sample buffer, heat‐denatured at 95 °C for 5 min, and loaded onto the gels. The molecular size standard was the peqGOLD protein‐marker V (Peqlab, Erlangen, Germany).

For the protein transfer to FluoroTrans W membranes (Pall, Port Washington, WI, USA), the mini VE vertical electrophoresis system (GE Healthcare, Munich, Germany) was used. Subsequently, the membranes were blocked with 5% low‐fat powdered milk (Roth, Karlsruhe, Germany) in TBST (blocking buffer) on a rocker for 1 h. The following primary antibodies were applied. From Cell Signaling Technology (Danvers, MA, USA): anti‐alpha‐Tubulin antibody, rabbit monoclonal (clone 11H10), dilution 1 : 10 000; anti‐CYR61 antibody rabbit monoclonal (clone D4H5D), dilution of 1 : 2000; anti‐Integrin β3 antibody, rabbit monoclonal (clone D7X3P), dilution 1 : 2000. From BD Biosciences (Heidelberg, Germany): Anti‐Integrin αv antibody, mouse monoclonal (clone 21/CD51), dilution 1 : 500. For the dilution of the primary antibodies, blocking buffer was used and the membranes were incubated with the primary antibody dilution buffer on a roller at 4 °C over night. The secondary antibodies conjugated with horseradish peroxidase (all from DAKO, Glostrup, Denmark) were diluted at a 1 : 1000 dilution in blocking buffer and applied onto the membranes for 90 min at room temperature. The signals were detected by using the Signal Fire ECL Reagent (Cell Signaling Technology) and X‐ray films (Agfa Health Care, Mortsel, Belgium) according to the manufacturer’s instructions. X‐ray films were digitized using the GS‐700 imaging densitometer (Bio‐Rad). Each sample was analyzed in three biological triplicates.

### 2.4 ELISA on cell lines

Cell culture supernatant of cell lines was clarified by centrifugation at 2500 ***g*** for 15 min. One microliter of cell culture supernatant was applied for ELISA. For whole cell lysate of cell lines, the cells were lysed with LPIP buffer (140 mm NaCl, 50 mm Tris HCl, pH 7.5, 1 mm EDTA, 0.05% NP40, 10% glycerol) and 10 µg the cell lysates was applied. The diluent was PBS (Gibco/Life Technologies, Paisley, UK). The protein concentration was determined using the Pierce BCA Protein Assay Kit (Pierce) according to the manufacturer’s instructions and using BSA as the standard.

For the analysis of the CYR61 secretion kinetics in H1993 and LC‐M1, the cells were cultured in T25 cm^2^ cell culture flasks.

The cells were allowed to settle on the flasks for 48 h. Next, the culture medium was changed and left on the cells for the indicated duration of time. After that, the cell number in the culture flasks was determined, the volume of the cell culture medium was determined, and the cell culture medium was analyzed for CYR61 by ELISA. One microliter of cell culture supernatant was applied for time points 1 h, 3 h, and 5 h. For the time points 5 min, 15 min, and 30 min, 10 µL of cell culture supernatant was applied.

The cell numbers were determined by seeding the cell lines in six‐well plates in parallel. For each well, 15 000 to 25 000 cells were seeded and allowed to grow for 24 h. The starting cell number was determined by counting the cell number of one well. Detachment of the cell was done by trypsinization and transfer to a Neubauer counting chamber. Dead cells were detected by using the vital stain trypan blue (Sigma‐Aldrich, St. Louis, MO, USA). The cell number was determined for nine squares, and the cell number per well was calculated for these values. The obtained cell number was taken as the starting cell number (*t* = 0 h) for the individual experiment. For the other time points, cells were cultivated in additional wells in parallel and processed as described for each experiment. After the appropriate time points, the cells were harvested and counted.

### 2.5 Generation of calibration curves for CYR61

For the generation of calibration curves, recombinant Cyr61 was spiked into the plasma of healthy donors who are negative for Cyr61. For all wells, unspecific background was eliminated by subtraction of OD values of wells containing cell culture medium. The conversion of the OD450 values from the standard curve to Cyr61 amounts was done by linear regression analysis using originpro version 9.6.5.169 (OriginLab Corporation, Northampton, MA, USA) (Fig. S1). For the analysis of cell lines, recombinant Cyr61 was spiked into cell culture medium (Fig. S2).

Within‐day variations of the ELISA were determined by the analysis of a dilution series of cell culture supernatant of MDA‐MB‐231 as Cyr61‐positive samples. As Cyr61 negative samples, cell culture supernatant of MCF‐7 was analyzed. Two different plates were analyzed on each day in a time series. For each measurement value of one day, the average value and the standard deviation were determined. The variation coefficient was determined by standard deviation divided by the average value (Table S1).

### 2.6 Immunocytochemical staining

The subcellular localization of CYR61 was analyzed by immunocytochemical double staining of CYR61 with keratin. The cell line HCC‐366 was seeded in chamber slides (Nunc Lab‐Tek Chamber Slide system, 2‐Well Permanox Slide, Thermo Scientific) and cultured for 2 days (20 000 cells per well). The cell culture medium was removed and the cells were washed with PBS, followed by fixation of the cells by 2% paraformaldehyde for 10 min and a washing step using PBS (3 min). Cell permeabilization was done using 0.1% Triton X‐100 in PBS for 10 min. Remaining Triton X‐100 was removed by two washing steps with PBS for 3 min each. Unspecific binding sites were blocked using 10% AB‐serum (Biotest, Dreieich, Germany) in PBS for 20 min. As primary anti‐CYR61 antibody, the antibody H2 (Santa Cruz Biotechnology, Dallas, TX, USA) was used in a dilution of 1 : 100 in 10% AB‐serum/PBS. The primary anti‐Cyr61 remained for 60 min on the cells. Next, by three washing steps with PBS for 3 min (each step), residual antibody was removed. Application of a secondary rabbit anti‐mouse fluorochrome antibody (546 nm, Molecular Probes, Eugene, OR, USA) was used for the revealing of the anti‐CYR61 antibody. The secondary antibody was used in a 1 : 200 dilution. The diluent was 10% AB‐serum/PBS, and the antibody was applied to the cells for 45 min. Unbound secondary antibody was removed by three washing steps with PBS.

For the detection of keratin, an antikeratin‐specific antibody combination was applied consisting of the anti‐pan‐keratin antibody (mouse monoclonal, clone AE1/AE3; Thermo Scientific) and the anti‐pan‐keratin antibody C11 (mouse monoclonal; Cell Signaling Technology). To minimize interference with the anti‐CYR61 staining, the antikeratin antibodies were applied as a direct‐conjugate with Alexa Fluor 488. The antibodies were diluted with 10% AB‐Serum in PBS applying a 1 : 100 dilution for C11 and a 1 : 80 dilution for the AE1/AE3 cocktail. The nuclei were stained using the DNA‐specific probe 4',6‐diamidino‐2‐phenylindole (Dapi, Sigma, Munich, Germany). Dapi was applied in a final concentration of 1 µg·mL^−1^ and was incubated for 60 min on the cells. Residual Dapi was removed by three washing steps. After mounting of the cells by ProLong Gold Antifade Mountant (Thermo Scientific), the cells were analyzed using the microscope Axioplan 2 (Carl Zeiss AG, Oberkochen, Germany).

### 2.7 Study population and blood collection

Eighty‐seven LC patients were recruited at the Helios Clinic Emil von Behring, Berlin, Germany. All participants provided written informed consent. The study was designed according to the rules guarding patient privacy and with the approval from the ethics committee of the Ruhr University Bochum (reference number 3217‐08). Peripheral blood of patients with diagnosed LC was drawn using 9.0 mL S‐Monovette K2‐EDTA gel tubes (Sarstedt, Nümbrecht, Germany). Plasma was obtained by centrifugation at 2000 ***g*** for 10 min at room temperature within 30 min after phlebotomy. Plasma was frozen immediately at −20 °C. Samples were regularly transported to the Institute for Prevention and Occupational Medicine of the German Social Accident Insurance, Institute of the Ruhr University Bochum (IPA), aliquoted, and stored at −80 °C. For the analysis of CYR61, frozen samples were transported to the Department of Tumor Biology, University Medical Center Hamburg‐Eppendorf.

In total, the study included 150 healthy controls. The blood of 140 healthy controls was obtained from the Department of Transfusion Medicine, University Medical Center Hamburg‐Eppendorf. A history of cancer was excluded as part of the mandatory entry procedure for blood donors. The human investigations were performed according to the Helsinki rules after approval was obtained by the ethics committee of the Medical Association Hamburg (reference number PV5392). From all patients, written informed consent was obtained. Peripheral blood of healthy controls was drawn using 9.0 mL S‐Monovettes K3E (Sarstedt). Plasma was obtained by centrifugation at 2000 ***g*** for 20 min at 4 °C. Plasma was subsequently aliquoted and stored at −80 °C until analysis of CYR61. Additionally, plasma samples from ten healthy current smokers were purchased from BioCat (Heidelberg, Germany) and included into the group of healthy controls. Samples were stored at −80 °C.

We carried out a retrospective study. Eligibility criteria were as follows: 18 years and older; men/women. LC samples were collected between September 2010 and November 2016 and healthy controls between July 2017 and March 2018. The identification of the LC patients was done by prior diagnosis and the cases formed a consecutive series. As a reference standard, healthy male and female individuals with an age of more than 50 years were selected (age‐matched to the patients). Further information on healthy controls is not available.

### 2.8 Enzyme‐linked immunosorbent assay (ELISA)

For the sandwich ELISA, the wells were first coated with the catching antibody, which was the anti‐CYR61 antibody H2 (Santa Cruz Biotechnology). The antibody was allowed to bind to the surface of the wells 4 °C overnight with gentle agitation. Unbound antibody was removed with three washing steps using phosphate‐buffered saline with 0.02% Tween‐20 (PBST). Next, unspecific binding was blocked with blocking buffer (5% nonfat dry milk in PBST) followed by three washing steps with PBST. For the application of the plasma samples, the wells were first filled with 95 µL of DMEM with 10% FCS followed by application of 5 µL of the plasma to each well. The assay was incubated for 2 h at room temperature with gentle agitation. Subsequently, three washing steps with PBST were performed and the anti‐CYR61 antibody H78 (Santa Cruz Biotechnology) was added to the wells. The incubation of this detecting antibody was performed at room temperature with gentle agitation for 2 h. Residual antibody was removed by three washing steps with PBST. For detection of CYR61, a polyclonal goat anti‐rabbit immunoglobulin antibody coupled with horseradish peroxidase (DAKO) was applied to each well. Unbound secondary antibody was removed by three washing steps with PBST. Chromogenic detection was performed by using 3,3´,5,5´ tetramethylbenzidine (TMB). TMB one component HRP microwell substrate was purchased from (Bethyl Laboratories, Montgomery, USA) fir 12 min followed by stopping the reaction by 100 µL of stop solution for TMB Substrates (ImmunoChemistry Technologies, Bloomington, IN, USA). For the detection of the extinction, the ELISA reader NanoQuant infinite M200 pro (Tecan, Männedorf, Switzerland) was used. The OD values were converted to CYR61 concentrations using recombinant and purified CYR61 protein from Abnova (Taipei, Taiwan) as a standard.

### 2.9 Statistical analysis

Arithmetic mean and standard deviation were used to describe the distribution of the CYR61 concentrations in the study groups. Groups were compared using the nonparametric Mann–Whitney *U*‐test or the Kruskal–Wallis test (with *P* < 0.05 as statistically significant). Furthermore, boxplots with median, interquartile range (IQR), and whiskers represent minimum and maximum display CYR61 concentrations. Sensitivity and specificity were determined from receiver operating characteristic (ROC) curves illustrating the performance of CYR61 to discriminate the studied groups. The area under the curve (AUC) was determined with its 95% Wald confidence interval (CI). Biomarker cut‐offs were determined with maximum Youden's index (YI, YI = sensitivity + specificity − 100) or at a predefined specificity of 100%. The parameters AUC, sensitivity and specificity were used for the determination of diagnostic accuracy. Statistical analyses were performed either using sas, version 9.4 (SAS Institute Inc., Cary, NC, USA) or originpro version 9.6.5.169 (OriginLab Corporation). Figures were generated with prism 7 (GraphPad Software, La Jolla, CA, USA). All data analyzed during this study are included in Table S2.

## 3 Results

### 3.1 Detection of CYR61 in cell lines and peripheral blood mononuclear cells (PBMC)

**Fig. 1:**
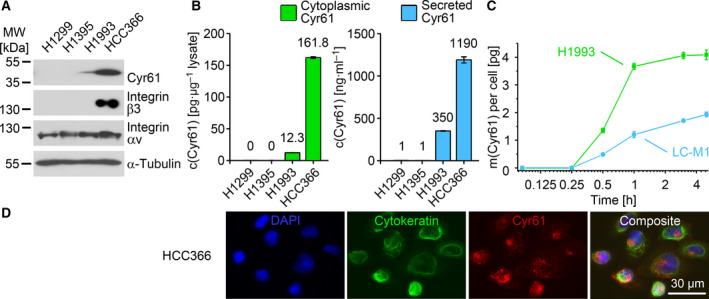
Detection of CYR61 and its secretion in lung cancer cells. All experiments *n* = 3. (A) Identification of CYR61‐positive lung cancer cell lines by western blot. (B) Quantification of the cytoplasmic CYR61 concentrations (diagram left) and secreted CYR61 (diagram right) by ELISA. The values are the average of three independent experiments, and vertical error bars show the standard deviation. (C) CYR61 secretion rates of H1993 and LC‐M1 calculated as mass of secreted CYR61 in picogram per cell. (D) Subcellular localization of CYR61 in the non‐small lung cancer cell line HCC‐366. The composite image is an overlay of the DAPI, cytokeratin, and CYR61 signals. c, concentration; CYR61, cysteine‐rich angiogenic inducer 61; h, hour; m, mass; MW, molecular weight; *n*, number of biologically independent replicates.

Starting with cell line models, we detected cytoplasmic CYR61 in LC cell lines by Western Blot (Fig. 1A). HCC‐366 was strongly positive for CYR61, whereas lower concentrations were detected in H1993. No signal could be detected for H1299 and H1395.

One of the preferred receptors of CYR61 is the integrin αv/integrin β3 heterodimer. These two integrins were present in the strongly CYR61‐positive LC cell line HCC‐366, while the other cell lines lacked detectable amounts of integrin β3.

As prerequisite for the subsequent measurement in clinical samples, we measured cytoplasmic and secreted CYR61 concentrations in LC cell lines by ELISA (Fig. 1B). A cytoplasmic CYR61 amount of 161.8 pg per µg cell lysate was detected for HCC‐366 and 12.3 pg per µg cell lysate for H1993. No signals were detected in lysates from H1299 and H1395. For secreted CYR61, a concentration of 1190 ng·mL^−1^ cell culture supernatant was detected for HCC‐366 and 350 ng·mL^−1^ for H1993. For H1299 and H1395, 1 ng·mL^−1^ CYR61 was detected.

Next, we investigated the CYR61 secretion kinetics (Fig. 1C). We replaced the cell culture medium from flasks with confluent cells by fresh medium and determined the released CYR61 concentration in the cell culture supernatant. Besides H1993, we analyzed the cell culture supernatant of the LC bone marrow DTC cell line LC‐M1. We observed that H1993 quickly released CYR61 into the medium reaching a plateau with a CYR61 amount of about 4 pg per cell after 1 h. For LC‐M1, a more continuous CYR61 secretion was observed, reaching a CYR61 concentration of approximately 2 pg per cell after 5 h.

We then determined the subcellular distribution of CYR61 in HCC‐366 and observed that CYR61 was distributed as cytoplasmic granules and was frequently found adjacent to the nucleus (Fig. 1D). Keratin staining was performed to visualize the cell shape.

### 3.2 Characteristics of the clinical study

**Fig. 2:**
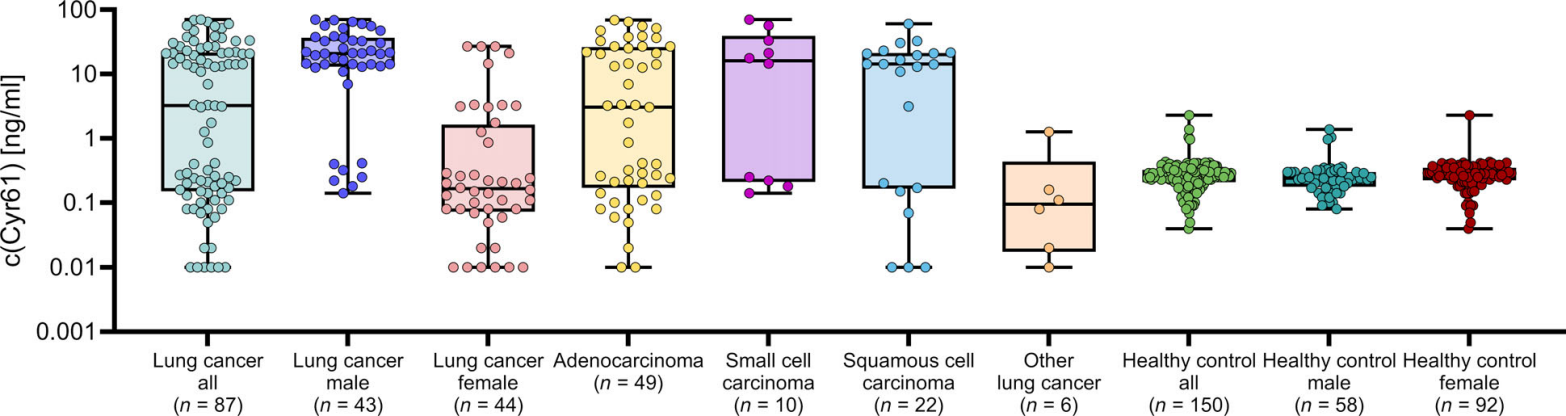
Distribution of CYR61 in the plasma of lung cancer patients and healthy controls divided into subgroups according to sex and histology. Horizontal bars display the median and the interquartile range. Lung cancer all patients *n* = 87; lung cancer male patients *n* = 43; lung cancer female patients *n* = 44; adenocarcinoma patients *n* = 49; small cell carcinoma patients *n* = 10; other lung cancer patients *n* = 6; healthy control all participants *n* = 150; healthy control male participants *n* = 58; healthy control female participants *n* = 92. For statistical analysis, values refer to Table 1. c, concentration; CYR61, cysteine‐rich angiogenic inducer 61; *n*, number of subjects.

We investigated 43 male and 44 female LC patients, comprising 49 ADC, 22 SCC, 10 SCLC, and 6 other LCs (Table 1). The healthy controls consisted of 58 men and 92 women. The distribution of circulating CYR61 in the plasma is presented in Fig. 2. CYR61 concentrations in plasma were elevated in all LC patients (13.7 ± 18.6 ng·mL^−1^) compared with all healthy controls (0.29 ± 0.22 ng·mL^−1^; *P* = 0.0003). Analysis of sex‐specific differences revealed that CYR61 concentrations were higher in male LC patients (25.1 ± 20.0 ng·mL^−1^) compared with male healthy controls (0.28 ± 0.22 ng·mL^−1^; *P* ˂ 0.0001). CYR61 concentrations were lower in female LC patients (2.6 ± 6.6 ng·mL^−1^) compared with male LC patients but they remained elevated compared with CYR61 concentrations in healthy women (0.30 ± 0.23 ng·mL^−1^, *P* = 0.0054).

**Table 1:** Clinico‐pathological parameters of lung cancer patients and concentrations of CYR61 in the plasma of lung cancer patients stratified by sex.

	Total			Men			Women		
	*n*	c(CYR61) [31]^a^	*P*‐value^b^	*n*	c(CYR61) [ng·mL^−1^]^a^	*P*‐value^b^	*n*	c(CYR61) [ng·mL^−1^]^a^	*P*‐value^b^
All	87	13.7 ± 18.6		43	25.1 ± 20.0		44	2.6 ± 6.6	
Age at the time of blood sampling									
< 50	5	4.5 ± 9.8	0.3125	1	22.0	0.3517	4	0.1 ± 0.1	0.0062
50–< 60	16	14.6 ± 19.7		7	30.2 ± 20.9		9	2.4 ± 4.8	
60–< 70	30	12.5 ± 20.2		11	28.5 ± 25.3		19	3.2 ± 7.5	
70–< 80	30	17.6 ± 18.5		22	23.9 ± 17.6		8	0.5 ± 0.8	
≥ 80	6	6.3 ± 10.5		2	3.6 ± 4.8		4	7.7 ± 12.9	
Sex									
Male	43	25.1 ± 20.0	0.0084						
Female	44	2.6 ± 6.6							
Smoking status									
Never	9	0.3 ± 0.6	0.0052	0		0.0724	9	0.3 ± 0.6	0.7389
Former	58	17.6 ± 20.3		33	27.8 ± 20.7		25	4.2 ± 8.5	
Current	20	8.5 ± 13.3		10	16.4 ± 15.2		10	0.6 ± 1.0	
Histology									
Adenocarcinoma	49	13.5 ± 19.3	0.0476	19	29.0 ± 21.8	0.4686	30	3.6 ± 7.8	0.1084
Small cell carcinoma	10	21.5 ± 25.2		10	21.5 ± 25.2		0		
Squamous cell carcinoma	22	14.4 ± 14.7		14	22.4 ± 12.7		8	0.5 ± 1.1	
Others	6	0.3 ± 0.5		0			6	0.3 ± 0.5	
Stage									
IIB	4	9.0 ± 10.8	0.8261	2	17.9 ± 5.2	0.9031	2	0.1 ± 0.04	0.0779
IIIA	13	18.3 ± 17.5		9	26.4 ± 14.8		2	0.1 ± 0.09	
IIIB	11	14.4 ± 20.4		6	22.4 ± 24.4		5	4.9 ± 9.2	
IV	59	12.9 ± 19.1		26	25.9 ± 21.8		33	2.7 ± 6.8	
T‐stage									
1	3	20.4 ± 34.8	0.6340	2	30.4 ± 42.7	0.7870	1	0.2	0.0273
2	28	10.8 ± 16.2		13	20.8 ± 18.2		15	2.2 ± 6.9	
3	23	13.2 ± 14.3		14	21.7 ± 12.2		9	0.1 ± 0.1	
4	33	15.9 ± 21.9		14	31.8 ± 24.9		19	4.2 ± 7.8	
Nodal status									
0	4	5.4 ± 6.7	0.6613	2	10.6 ± 5.2	0.5673	2	0.2 ± 0.1	0.1564
1	11	17.9 ± 15.1		7	20.5 ± 15.5		4	13.5 ± 15.6	
2	41	14.3 ± 20.4		21	27.5 ± 21.3		20	0.4 ± 1.0	
3	31	12.5 ± 18.4		13	26.0 ± 21.6		18	2.8 ± 5.7	
Metastasis									
0	28	15.5 ± 17.7	0.1673	17	24.0 ± 17.5	0.3458	11	2.2 ± 6.4	0.0135
1a	20	18.3 ± 23.1		10	32.7 ± 24.5		10	3.9 ± 8.2	
1b	39	10.1 ± 16.4		16	21.6 ± 19.6		23	2.2 ± 6.2	

a Arithmetic mean ± standard deviation.

b Kruskal–Wallis test.

### 3.3 Correlation of the CYR61 concentrations with clinical parameters

We analyzed potential correlations of CYR61 with clinico‐pathological parameters (Table 1). We found an association of the CYR61 concentrations with sex (*P* = 0.0084), smoking status (*P* = 0.0052), and cancer histology (*P* = 0.0476).

Since we found large differences between CYR61 concentrations in men and women, it seems reasonable to consider both sexes separately. In men, smoking status showed a marginal effect on CYR61 concentration (*P* = 0.0724). In women, CYR61 concentrations differed between age groups (*P* = 0.0062), T‐stage (*P* = 0.0273), and metastasis status (*P* = 0.0135). In contrast, the found association between CYR61 concentration and cancer histology in the total study population might rather be an expression of the sex‐specific CYR61 difference (*P* = 0.0476). For the other clinical parameters, no statistically significant association was observed.

### 3.4 Receiver operating characteristic (ROC) analyses of CYR61 concentrations in plasma

**Fig. 3:**
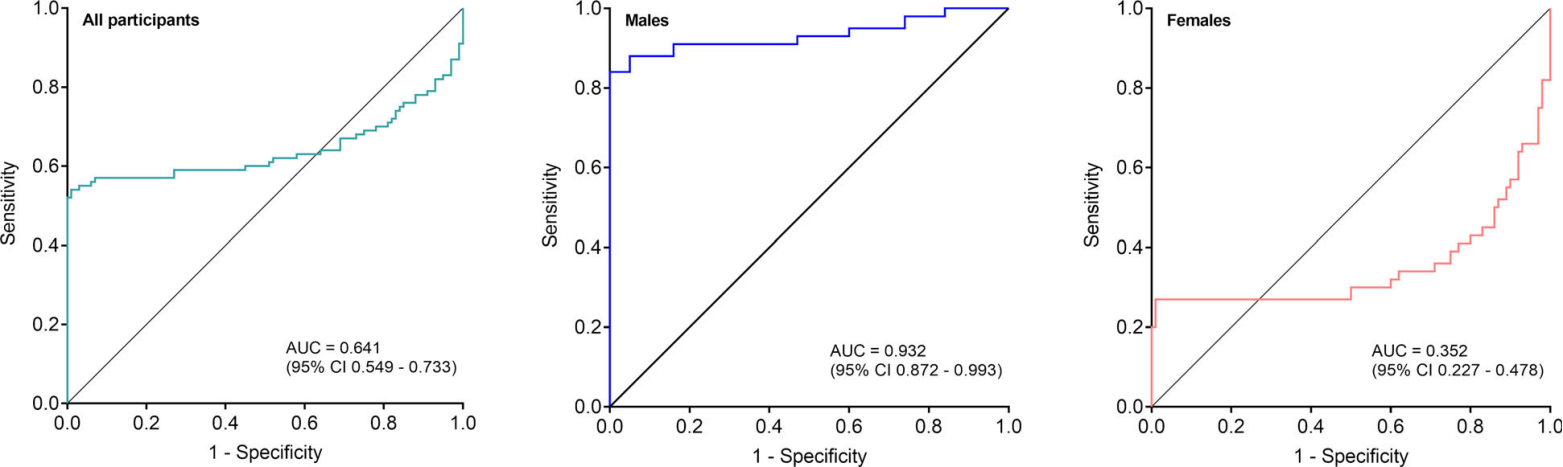
Receiver operating characteristic (ROC) curves of CYR61 in all lung cancer patients and healthy controls and stratified by sex. All participants: lung cancer patients *n* = 87; healthy controls *n* = 150. Males: male lung cancer patients *n* = 43; male healthy controls *n* = 58. Females: female lung cancer patients *n* = 44; female healthy controls *n* = 92. For statistical analysis, values refer to Table 2. AUC, area under the curve; CI, confidence interval; *n*, number of subjects.

Results of the ROC analyses of CYR61 concentrations in plasma are presented in Fig. 3 and Table 2. For the comparison of all LC patients *vs*. all healthy controls, we obtained an AUC of 0.641 (95% CI 0.549–0.733). Using the maximum YI, a sensitivity of 54% at 99% specificity was revealed. Comparing male LC patients *vs*. male controls, an improved AUC of 0.932 (95% CI 0.872–0.993) with a sensitivity of 84% at 100% specificity was obtained (maximum YI). In contrast, an inferior performance was observed for females (AUC = 0.352 (95% CI 0.227–0.478); sensitivity: 27%; specificity: 99%; maximum YI).

**Table 2:** Parameters for the specified study groups after CYR61 detection in plasma.

Study group	Sex	*n*	c(CYR61) [ng·mL^−1^]^a^	*P*‐value^b^	Binary classifier			
					AUC^c^	95% CI^d^	Sensitivity [%] / specificity [%]^e^ / marker cutoff [ng·mL^−1^] at maximum Youden's index	Sensitivity [%] at 100% specificity [%] / marker cutoff [ng·mL^−1^]
Lung cancer	All	87	13.7 ± 18.6			0.549		
*vs*.				0.0003	0.641	‐	54 / 99 / 1.27	52 / 3.07
Healthy control	All	150	0.29 ± 0.22			0.733		
Lung cancer	Male	43	25.1 ± 20.0			0.872		
*vs*.				<0.0001	0.932	‐	84 / 100 / 6.97	84 / 6.97
Healthy control	Male	58	0.28 ± 0.22			0.993		
Lung cancer	Female	44	2.6 ± 6.6			0.227		
*vs*.				0.0054	0.352	–	27 / 99 / 0.86	20 / 3.07
Healthy control	Female	92	0.30 ± 0.23			0.478		

^a^ Arithmetic mean ± standard deviation.

^b^ Mann–Whitney *U*‐test.

^c^ Area under the curve.

^d^ Confidence interval.

## 4 Discussion

We investigated the potential of circulating CYR61 in plasma as a biomarker for the detection of LC. Firstly, we confirmed by western blot and immunostaining that LC cells are able to express cellular CYR61. Cell line HCC‐366 showed strong expression of cellular CYR61 together with strong expression of integrin α_v_β_3_, which is known as direct receptor for CYR61 [46]. It was shown that CYR61 stimulates direct chemotaxis [47] and Tsai *et al*. [33] proposed that CYR61 can mediate tumor growth and angiogenesis in either an autocrine or paracrine manner through its binding to the α_v_β_3_ integrin receptor. These findings could possibly explain the difference in expression level between HCC‐366 and H1993 since H1993 cell line showed no expression of integrin β_3_ and weak expression of cellular CYR61 in our experiments. Cell lines H1299 and H1395 showed no expression of both, CYR61 and integrin β_3_. Subsequent ELISA analysis showed that LC cells are also secreting CYR61 protein with HCC‐366 showing the highest level of secretion.

After demonstrating *in vitro* that LC cells express and secrete substantial amounts of CYR61, we also detected circulating CYR61 concentrations in plasma samples from LC patients. Our results revealed a significant positive association of the CYR61 plasma concentration with smoking status; current and former smokers had elevated CYR61 concentrations compared with never smokers. The higher concentration in current smokers is consistent with the view that CYR61 is a tissue stress sensor [48, 49, 50]. However, the rather small sizes of the study groups and an unequal distribution of current, former, and never smokers, as well as all never smokers in our cohort were women, could lead to artificially decreased concentration of CYR61 in the group of never smokers and therefore lead to misconclusions. In line with this, we could not find statistically significantly different CYR61 concentrations depending on smoking status in the group of female LC patients. Therefore, the role of smoking status and sex on CYR61 concentrations needs to be further elucidated.

Additionally, we found an association between CYR61 concentrations and histological subtype but this observation might be based on an uneven distribution of histology between sexes. For example, the study group for SCLC comprises only of male participants which could lead to an artificial increase in CYR61 concentration when both sexes are combined together. Hence, the association between CYR61 concentration and histological subtype should be further investigated. However, this result is consistent with a previous report from Chen *et al*. [21]. On the other hand, Mori *et al*. [24] reported no correlation between CYR61 expression level and histological subtype. It should be noted that in both works tissue samples have been analyzed, whereas we analyzed circulating CYR61 in plasma. Therefore, future studies are needed also in order to compare the expression of CYR61 in tissues with circulating CYR61.

Our key finding was the high sensitivity and specificity of circulating CYR61 concentrations for detection of LC in men. Stratification by sex revealed a sensitivity of 84% for men compared with only 27% for women at high specificities of 100% and 99%, respectively. The mechanism behind this sex‐specific difference remains unclear. However, previous studies have reported sex‐specific actions of other biomarkers in LC possibly due to endocrine difference involving the estrogen signaling axis [51, 52]. It has been reported previously that estrogen and CYR61 are promoting cancer progression through the Hippo signaling pathway [29, 53, 54]. Therefore, it is possible that cancer progression is driven via the effect of estrogen in females while in men it is rather driven by CYR61 instead of estrogen.

Previous immunohistochemical analyses of CYR61 expression in LC tissue have not revealed significant sex‐specific differences [21, 24]. Besides technical reasons (e.g., choice of antibodies, fixation of tissue), this might be explained by the assumption that CYR61 secretion into the blood is not only determined by the cytoplasmic protein content of CYR61 but also involves other factors with an impact on the release of CYR61 into the blood. This hypothesis is in line with our recent report on CYR61 secretion in breast cancer [55], and it might stimulate future experimental work. Comparing CYR61 with other blood biomarkers of the liquid biopsy family, such as CTCs, ctDNA, and miRNA, showed that CYR61 has rather higher sensitivity and specificity for the detection of LC (Table 3). Moreover, CYR61 protein is relatively stable in plasma [56] and CYR61 concentrations can be easily measured by ELISA at low costs with very small amounts of plasma. Additionally, ELISA is an established method useful for screening routine in a central laboratory. On the other hand, no isolation method for the analysis of LC‐derived CTCs, ctDNA, or miRNA has been standardized yet and isolation methods that are currently being used are either time‐consuming or are suffering from large yield variations, low sensitivity, low purity, or low recovery rate [57, 58, 59] compared with isolation of plasma and detection of secreted proteins. However, the advantage of the use of other analytes compared with secreted proteins, such as CYR61, is further molecular analysis after detection.

**Table 3:** Comparison of the accuracy of different blood tests used for the detection of lung cancer.

Analyte	Patient criterion	Sensitivity	Specificity	Study description	Reference
CTCs	Inclusion criteria: NLST‐UPSTF criteria plus COPD defined as persistent respiratory symptoms and fixed airflow limitation with a postbronchodilator FEV1/forced vital capacity < 0.7 Exclusion criteria: any cancer, other than basocellular skin carcinoma, detected within the previous 5 years. Full exclusion criterion is reported in Leroy *et al*. [60]	26.30% (95.00% CI 11.80%–48.80%)	96.20% (95.00% CI 94.40%–97.50%)	Participants underwent three screenings at 1‐year intervals. Each screening round consisted of a clinical examination, a LDCT, and a blood test to detect CTCs (ISET Rarecells; Rarecells Diagnostics, Paris, France). The primary endpoint of the study was the diagnostic performance of CTC detection as a biomarker for diagnosis of lung cancer screening, could CTC detection act as a screening tool? For this purpose, the detection of CNHC‐malignant and CNHC‐uncertain was considered as positive for cancer diagnosis	[13]
ctDNA	Inclusion criteria: (i) NSCLC stage III‐IV (ii) the selected patients were diagnosed both histopathologically and cytologically (iii) the data on TP, TN, FP and FN were fully reported to construct 2 × 2 table (iv) the EGFR mutation was detected Exclusion criteria: (i) Peripheral blood and tumor tissues were not paired (ii) the case sample number was ˂ 10 in the case series studies (iii) the study did not clarify the tumor stage and the data of advanced NSCLC could not be extracted	70.00% (95.00% CI 63.00%–75.00%)	98.00% (95.00% CI 96.00%–99.00%)	Meta‐analysis of the value of peripheral blood ctDNAs in detection of EGFR mutations in patients with advanced NSCLC. A total of 32 studies were finally included in the meta‐analysis	[61]
miRNA	LC patients I‐IV stage (NSCLC, SCLC) (*n* = 606) Control group (*n* = 2440): patients with other lung diseases, patients with other diseases not affecting lungs, unaffected control participants Inclusion criteria: symptomatic patients, available definitive clinical diagnosis	82.80% (95.00% CI 81.50%–84.10%)	93.50% (95.00% CI 93.20%–93.80%)	Signature of 15 miRNA was identified to distinguish patients diagnosed with lung cancer from all other individuals (patients with other lung diseases, patients with other diseases not affecting lungs and unaffected control participants)	[62]
CYR61 protein (in men)	LC patients II‐IV stage Inclusion criteria: ≥ 18 men Control group: healthy male individuals with an age ˃50 years (age‐matched to the patients)	84.00% (95.00% CI 70.40%–92.69%)	100.00% (95.00% CI 95.50%–100.00%)	The identification of the lung cancer patients was done by prior diagnosis	Our study

CI, confidence interval; CNHC‐malignant, circulating nonhematological cells with malignant features; CNHC‐uncertain, circulating nonhematological cells with uncertain malignant features; CTC, circulating tumor cells; ctDNA, circulating tumor DNA; FN, false negative; FP, false positive; TN, true negative; TP, true positive.

Limitation of this study is that our study population consisted of LC patients at advanced stage, whereas samples from individuals with pulmonary nodules or early‐stage LC samples were missing. However, for the detection of LC at early stages, CYR61 should be validated in plasma samples taken before clinical diagnosis using a large study with a prospective design.

## 5 Conclusions

Our present pilot study suggests that circulating CYR61 protein has the potential to serve as a new biomarker for the detection of LC in men. However, this proof‐of‐principle finding needs to be verified in larger study groups of LC patients, including nontumorous lung diseases which could lead to false‐positive signals since it was previously reported that CYR61 is elevated in asbestos‐associated diseases as well [31]. Within the context of LDCT screening, the performance of CYR61 for the use in screening procedures needs to be validated in appropriate prospective studies. Additionally, CYR61 is significantly elevated in female LC patients but sensitivity and specificity for CYR61 are too low for the improvement of the detection of lung cancer in women.

## Conflict of interest

KB and KP are inventors of patent applications including the use of CYR61 for early detection of breast cancer (No. EP1715 7020; No. PCT/EP2018/054052), otherwise no competing interests.

## Author contributions

All authors approved the final version manuscript. All authors are accountable for all aspects of the work. LA, KB, DGW, GJ, and KP involved in experimental design and manuscript writing. KB, LA, and AA performed experiments. KB, DGW, IR, TB, JK, MG, SP, and GJ involved in analysis of plasma samples & data analysis. SC carried out Statistics.

### Peer Review

The peer review history for this article is available at https://publons.com/publon/10.1002/1878‐0261.13099.

## Supporting information


**Fig. S1.** Calibration curves for the conversion of the OD values to the Cyr61 amount in this study.
**Fig. S2.** Calibration curves for the conversion of the OD values to the Cyr61 amount for the cell line experiments.

**Table S1.** Variation coefficients of Cyr61 levels in a time course.

**Table S2.** Clinical data of the study.

## Data Availability

All data are available within the main text and supporting material.
