# Supplementary material for: Blood‐based detection of lung cancer using cysteine‐rich angiogenic inducer 61 (CYR61) as a circulating protein biomarker: a pilot study
Source: Mol Oncol. 2021 Oct 3;15(11):2877–90. doi: 10.1002/1878-0261.13099 (PMC8564649; doi:10.1002/1878-0261.13099)
Supplement: Supplementary file 1 — Fig. S1. Calibration curves for the conversion of the OD values to the Cyr61 amount in this study. Fig. S2. Calibration curves for the conversion of the OD values to the Cyr61 amount for the cell line experiments. [file MOL2-15-2877-s002.docx]

Blood-based detection of lung cancer using cysteine-rich angiogenic inducer 61 (CYR61) as a circulating protein biomarker: a pilot study

Lucija Ačkar, Swaantje Casjens, Antje Andreas, Irina Raiko, Thomas Brüning, Maria Geffken, Sven Peine, Jens Kollmeier, Georg Johnen, Kai Bartkowiak, Daniel Gilbert Weber, Klaus Pantel

**Supplementary material**

**Supporting figures**


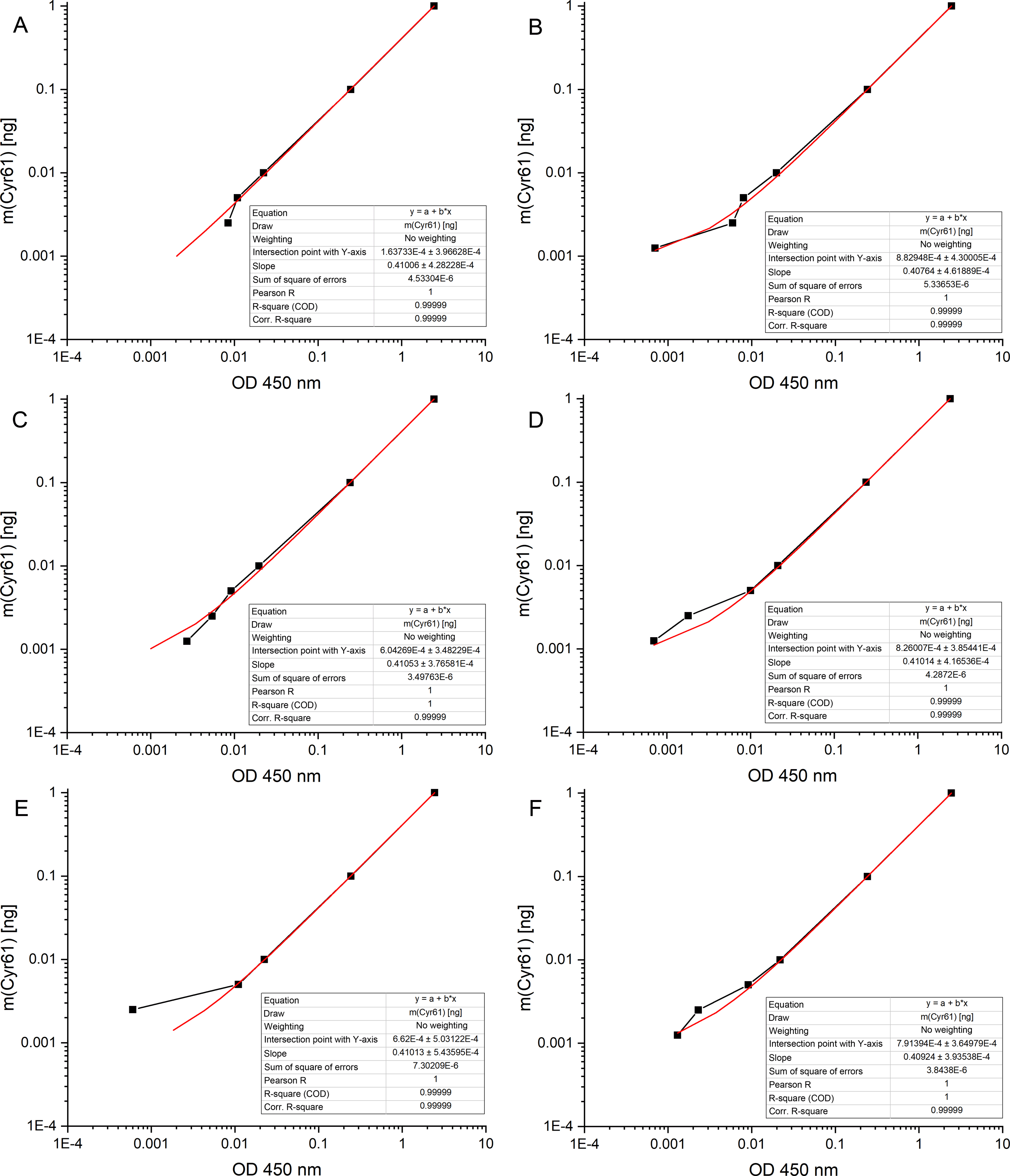


**Figure S1.** Calibration curves for the conversion of the OD values to the Cyr61 amount in this study. The calibration curves were done using linear fit. For a better representation, the curve progression is shown in double logarithmic scales in this image. The calibration curves were generated by spiking of recombinant Cyr61 into the plasma of Cyr61-negative healthy controls. A prior background subtraction was done by subtracting the OD value of wells that contained cell culture medium. A-C: calibration curves for the analysis of the lung cancer samples. D-E: calibration curves for the analysis of female healthy controls. F: calibration curve for the analysis of male healthy controls and healthy smokers. n=1. m=mass; ng=nanogram; OD=optical density; COD=coefficient of determination; n=biological and technical replicate


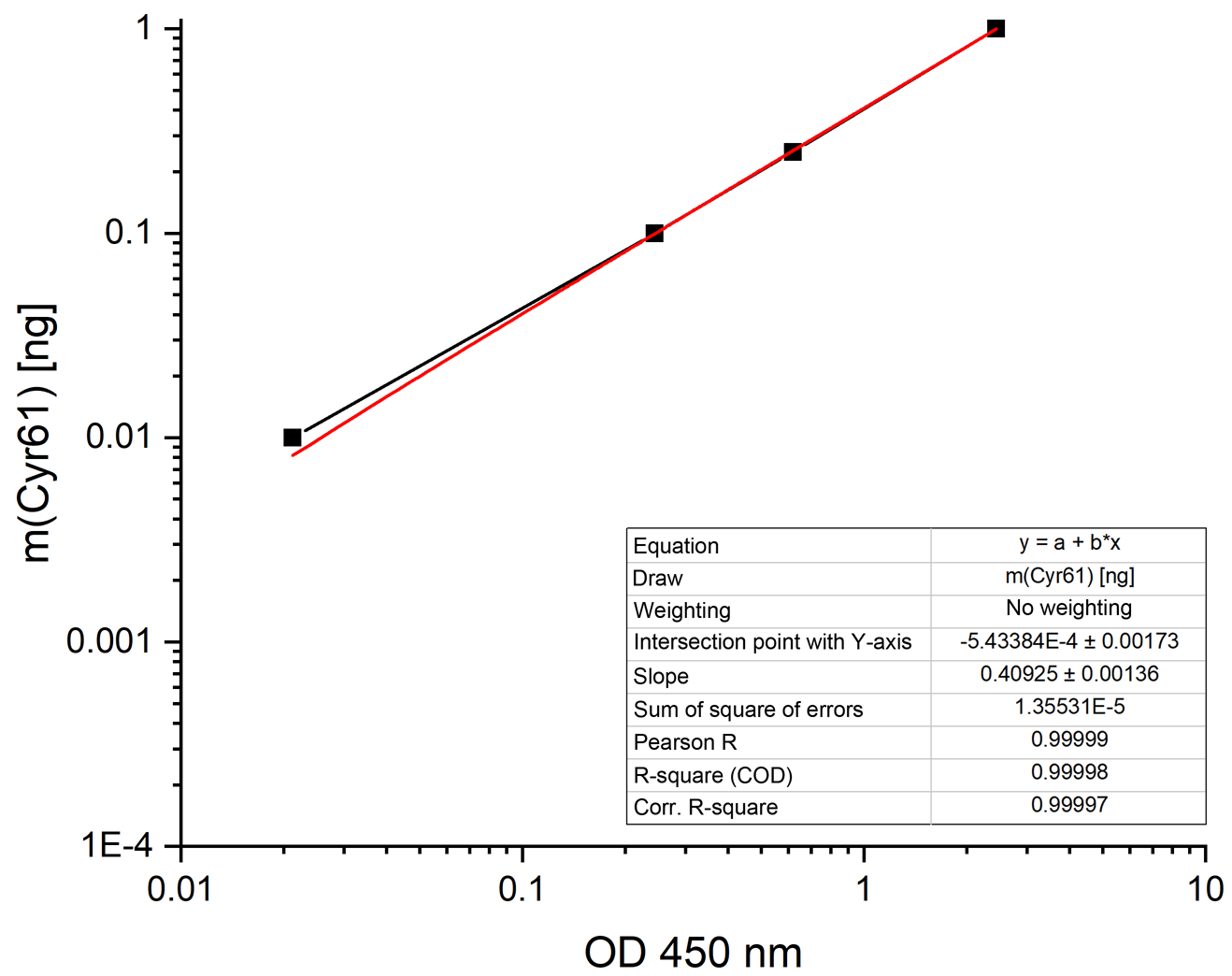


**Figure S2.** Calibration curves for the conversion of the OD values to the Cyr61 amount for the cell line experiments. The calibration curves were done using linear fit. For a better representation, the curve progression is shown in double logarithmic scales in this image. The calibration curves were generated by spiking of recombinant Cyr61 into cell culture medium. A prior background subtraction was done by subtracting the OD value of wells that contained cell culture medium without recombinant Cyr61. n=1. m=mass; ng=nanogram; OD=optical density; COD=coefficient of determination; n=biological and technical replicate
