# Supplementary material for: Blood‐based detection of lung cancer using cysteine‐rich angiogenic inducer 61 (CYR61) as a circulating protein biomarker: a pilot study
Source: Mol Oncol. 2021 Oct 3;15(11):2877–90. doi: 10.1002/1878-0261.13099 (PMC8564649; doi:10.1002/1878-0261.13099)
Supplement: Supplementary file 3 — Table S2. Clinical data of the study. [file MOL2-15-2877-s001.docx]

**Table S2.** Variation coefficients of Cyr61 levels in a time course. On each day two measurements of the Cyr61 concentrations were performed and the variation coefficient was determined for each measurement point. The cell culture supernatant of the Cyr61 negative cell line MCF-7 (2.5 µl) served as negative control (0 ng/ml) and different amounts of the cell culture supernatant of MDA-MB-231 (Cyr61 positive cell line) were analyzed. The values are the arithmetic means of the Cyr61 concentrations ± standard deviation [ng/ml].

| Day of the analysis | 0 ng/ml | 10.420 ng/ml | 1.042 ng/ml | 0.104 ng/ml | 0.052 ng/ml | 0.026 ng/ml |
| --- | --- | --- | --- | --- | --- | --- |
| 5 | 0.593 | 0.008  0.001  0.002  0.006  0.003  0.004  0.006  0.010  0.003  0.008  0.010  0.021  0.000  0.003 | 0.005  0.019  0.004  0.009  0.002  0.019  0.007  0.009  0.007  0.000  0.027  0.018  0.004  0.000 | 0.049  0.005  0.087  0.022  0.058  0.037  0.020  0.005  0.116  0.000  0.016  0.040  0.024  0.035 | 0.048  0.034  0.111  0.016  0.078  0.031  0.138  0.034  0.274  0.023  0.024  0.074  0.092  0.044 | 0.068 |
| 9 | 0.126 |  |  |  |  | 0.065 |
| 16 | 0.759 |  |  |  |  | 0.174 |
| 42 | 0.117 |  |  |  |  | 0.076 |
| 44 | 1.040 |  |  |  |  | 0.067 |
| 55 | 0.023 |  |  |  |  | 0.068 |
| 57 | 10.781 |  |  |  |  | 0.037 |
| 145 | 0.115 |  |  |  |  | 0.025 |
| 148 | 3.127 |  |  |  |  | 0.237 |
| 226 | 0.399 |  |  |  |  | 0.018 |
| 253 | 0.157 |  |  |  |  | 0.027 |
| 289 | -1.103 |  |  |  |  | 0.099 |
| 296 | -9.800 |  |  |  |  | 0.053 |
| 483 | -1.385 |  |  |  |  | 0.077 |
| all: arithmetic mean | 0.3536 | 0.0061 | 0.0093 | 0.0367 | 0.0729 | 0.0779 |
| all: standard deviation | 4.1663 | 0.0053 | 0.0083 | 0.0328 | 0.0685 | 0.0599 |
